# LC–MS/MS-Based Comparative Phytochemical Profiling and Multivariate (PCA) Analysis of Seed Extracts from *Trigonella foenum-graecum*, *Linum usitatissimum*, and *Panicum miliaceum* with Emphasis on Antioxidant and Antibacterial Activities

**DOI:** 10.3390/ijms27093947

**Published:** 2026-04-29

**Authors:** Aicha Boubker, Abdelmoula El Ouardi, Taha El Kamli, Adnane El Hamidi, Mohammed Kaicer, Faouzi Kichou, Khaoula Errafii, Rachid Ben Aakame, Aicha Sifou

**Affiliations:** 1 Laboratory of Materials, Nanotechnologies and Environment, Faculty of Sciences, Mohammed V University in Rabat, BP: 1014, Ibn Battouta Avenue, Rabat 10000, Morocco; boubkeraicha45@gmail.com (A.B.); a.elhamidi@um5r.ac.ma (A.E.H.); 2 Department of Microbiology of Water, Food and Environment, National Institute of Hygiene, BP: 769, Agdal, 27, Avenue Ibn Batouta, Rabat 10000, Morocco; abdoelouardi@yahoo.fr; 3 Laboratory of Anti-Doping Control, Hassan II Institute of Agronomic and Veterinary, BP: 6202, Madinat Al Irfane, Rabat 10000, Morocco; elkamlit@yahoo.fr; 4 Laboratory of Analysis, Geometry and Applications, Systems and Optimization, Ibn Tofail University, Campus of University, BP: 242, Kenitra 14000, Morocco; mohammed.kaicer@uit.ac.ma; 5 Department of Pathology and Veterinary Public Health, Hassan II Institute of Agronomic and Veterinary, BP: 6202, Madinat Al Irfane, Rabat 10000, Morocco; kichoufaouzi@gmail.com; 6 African Genom, University Mohammed VI Polytechnic Center, BP: 660, Hay Moulay Rachid, Benguerir 43150, Morocco; khaoula.errafii@um6p.ma; 7 Laboratory of Food Toxicology, National Institute of Hygiene, BP: 769, Agdal, 27, Avenue Ibn Batouta, Rabat 10000, Morocco; benakame@yahoo.fr

**Keywords:** *Trigonella foenum-graecum*, *Linum usitatissimum*, *Panicum miliaceum*, seed extracts, LC–MS/MS, phytochemical profiling, antioxidant activity, antibacterial activity, multivariate analysis

## Abstract

This study provides a comprehensive evaluation of the phytochemical composition, antioxidant capacity, antibacterial activity, and mineral content of *Trigonella foenum-graecum*, *Linum usitatissimum*, and *Panicum miliaceum* extracts obtained using aqueous, ethanolic, and methanolic solvents. An integrated analytical strategy combining LC–MS/MS-based metabolite profiling, mineral analysis, and multivariate statistical tools (PCA) was applied to investigate the relationships between chemical composition and biological activities. The ethanolic extract of *P. miliaceum* showed the highest total phenolic content (TPC: 157.438 ± 0.521 µg GAE/mg extract), whereas *L. usitatissimum* exhibited the strongest antioxidant activity (IC_50_ ≈ 65 µg/mL). *Trigonella foenum-graecum* displayed the most significant antibacterial activity, with a minimum inhibitory concentration (MIC) of 62.5 mg/mL against *Staphylococcus aureus*. LC–MS/MS analysis allowed the identification and structural characterization of more than twenty bioactive compounds through multiple reaction monitoring (MRM), including flavonoids, phenolic acids, and anthocyanins. Principal Component Analysis indicated that sample discrimination was mainly driven by solvent polarity rather than plant species, underlining the critical influence of extraction conditions on phytochemical profiles and associated bioactivities. These findings highlight the relevance of combining analytical and statistical approaches to better understand the interplay between plant origin, extraction conditions, and biological properties, and support the potential of these species as promising sources of nutraceutical and pharmaceutical compounds.

## 1. Introduction

Medicinal and edible plants have attracted increasing attention in recent years as valuable sources of bioactive natural compounds with diverse pharmacological properties [1]. Phenolic compounds, including flavonoids and tannins, are among the most important plant secondary metabolites, widely recognized for their antioxidant, antimicrobial, and anti-inflammatory properties. These compounds play a key role in protecting plant tissues against oxidative stress and microbial invasion, and they also contribute to human health by scavenging reactive oxygen species (ROS) and inhibiting the growth of pathogenic bacteria [2,3,4]. The growing interest in plant-derived bioactives is closely linked to the increasing demand for natural therapies and functional foods with health-promoting potential [5].

Numerous studies have highlighted the crucial roles of phenolic compounds in mitigating oxidative damage by scavenging reactive oxygen species and chelating metal ions [6], as well as their antimicrobial activity against various bacteria, including resistant strains [7]. For example, phenolic acids, flavonoids, and tannins from plant extracts have been shown to inhibit bacterial growth while providing strong radical-scavenging capacity [8]. In addition, flavonoids and other polyphenols are among the most extensively studied phytochemicals due to their diverse biological activities, including anticancer, cardioprotective, neuroprotective, and anti-inflammatory effects [9]. *Trigonella foenum-graecum* (fenugreek), *Linum usitatissimum* (flaxseed), and *Panicum miliaceum* (proso millet) are edible and medicinal plants that have been traditionally used across various cultures and are increasingly investigated for their pharmacological potential. Fenugreek, a leguminous plant belonging to the Fabaceae family, is well known for its antidiabetic, anti-inflammatory, and antimicrobial properties [10]. Previous studies have identified key bioactive compounds such as trigonelline, isoorientin, orientin, vitexin, and isovitexin in fenugreek seeds using LC–MS analysis [11]. *L. usitatissimum* (flaxseed) is recognized for its high content of omega-3 fatty acids, lignans, and phenolic compounds, which are associated with strong antioxidant and antibacterial activities [12]. Although *P. miliaceum* has received less attention, recent studies have demonstrated its potential as a source of phenolic acids and flavonoids with significant antioxidant activity [13]. Despite the growing body of research on plant-derived bioactive compounds, most studies have focused on individual aspects such as phytochemical composition or biological activity. However, integrative approaches combining phytochemical profiling, mineral composition, and antibacterial activity across different plant matrices remain limited. In particular, comparative studies evaluating the influence of extraction solvents on both chemical composition and biological properties are still scarce. These three species were selected to represent distinct plant matrices, namely a legume (*T. foenum-graecum*), an oilseed (*L. usitatissimum*), and a cereal (*P. miliaceum*). This selection enables a comparative evaluation of how botanical origin and matrix composition influence phytochemical profiles, mineral content, and biological activities. Given the nutritional and pharmacological importance of these species, this study aimed to provide a comprehensive comparison of their phytochemical composition and biological activities. To this end, an integrative approach was adopted, combining LC–MS/MS-based phytochemical profiling, mineral composition analysis, and biological activity evaluation (antioxidant and antibacterial), in order to better understand their molecular diversity and functional potential. Additionally, the influence of extraction solvents on bioactive compound recovery and biological efficacy was investigated. Multivariate statistical analysis (Principal Component Analysis, PCA) was employed to explore correlations between phytochemical composition, mineral content, and bioactivities. This study addresses the following questions: (i) how do extraction solvents influence phytochemical composition and biological activities, (ii) are there significant differences among the selected plant species, and (iii) can correlations be established between chemical composition, mineral content, and biological properties. This integrative framework provides new insights into the relationships between plant origin, extraction conditions, and biological properties.

## 2. Results and Discussion

### 2.1. Total TPC, TFC, TCT and Antioxidant Activity

The comparative analysis of *T. foenum-graecum*, *L. usitatissimum*, and *P. miliaceum* showed clear differences in their total phenolic content (TPC), flavonoid content (TFC), condensed tannins (TCT), and antioxidant activity depending on the solvent used for extraction in Table 1. Among the three, *T. foenum-graecum* had the highest phenolic concentration in its aqueous extract (73.26 µg GAE/mg E), while *P. miliaceum* stood out in the ethanolic extract with a very high TPC value (157.44 µg GAE/mg E). The higher phenolic content observed in the ethanolic extract of *P. miliaceum* may be attributed to the intermediate polarity of ethanol, which facilitates the extraction of both polar and moderately polar phenolic compounds. In contrast, *L. usitatissimum* showed lower TPC levels (9.98–10.80 µg GAE/mg E), but its antioxidant activity remained fairly stable across all solvents. This suggests that other bioactive compounds, such as lignans and polyunsaturated fatty acids, may also play a role in its radical-scavenging activity [12].

Compared with data from previous studies, the TPC of *P. miliaceum* in this work is much higher than that reported by [14], who found only 0–48 µg GAE/g of grain (around 0.048 µg GAE/mg). This big difference can be explained by variations in extraction methods, solvent polarity, and the concentration of the extract. Similarly, our *T. foenum-graecum* samples showed tannin levels (32–45 µg CE/mg extract) much higher than those reported by [15], who found only about 23–27 µg/g of dry matter. Again, this gap is mainly due to different units and extraction processes—our results are based on concentrated extracts, not whole-grain powder.

When looking at antioxidant activity, *L. usitatissimum* showed the strongest DPPH radical-scavenging power, with IC_50_ values between 65 and 71 µg/mL. It was followed by *T. foenum-graecum* (175–182 µg/mL) and *P. miliaceum* (283–301 µg/mL). These results show how both solvent type and plant composition affect antioxidant strength. Other studies have reported weaker activity for fenugreek extracts [16], for instance, found an IC_50_ of 366.52 µg/mL for methanolic extracts, while [17] also noted that solvent polarity strongly influences the antioxidant response. A review by [18] further confirmed that the antioxidant capacity of *T. foenum-graecum* is closely linked to its phenolic content, which supports our observations.

For *L. usitatissimum*, the IC_50_ values obtained here (70.94, 65.21, and 68.83 µg/mL for aqueous, ethanolic, and methanolic extracts) show much stronger antioxidant activity than the 297.39 µg/mL reported by [19] for an 80% ethanolic extract. The strong antioxidant activity observed in *L. usitatissimum* may not be solely attributed to phenolic compounds, but also to the presence of non-phenolic antioxidants such as lignans (e.g., secoisolariciresinol diglucoside), omega-3 fatty acids, and tocopherols, which are known to contribute significantly to its antioxidant potential [20]. Overall, these findings highlight how the type of solvent and the natural composition of each plant can significantly influence phenolic extraction and antioxidant behavior.

### 2.2. Determination of Phenolic Molecules by LC/MS/MS

Metabolomic profiling of the three studied species revealed distinct patterns of phenolic and flavonoid compounds Table 2, suggesting divergent yet potentially complementary metabolic pathways. It should be noted that the LC–MS/MS results are expressed as relative abundances rather than absolute concentrations, and therefore should be interpreted as semi-quantitative data reflecting comparative metabolite distribution. In *T. foenum-graecum*, the predominance of kaempferol-3-O-rutinoside (29.76%) and kaempferol-3-O-glucoside (20.43%) indicates an active flavonol biosynthetic pathway, which aligns with previous findings highlighting the antioxidant and anti-inflammatory potential of these compounds [21,22]. In *L. usitatissimum*, the exceptionally high content of L-phenylalanine (78.81%) suggests enhanced flux through the aromatic amino acid pathway, a critical precursor route for phenolic compound formation. This observation is consistent with recent reports emphasizing the role of phenylalanine as a key substrate in the phenylpropanoid pathway. Conversely, *P. miliaceum* exhibited a metabolite profile dominated by anthocyanins—particularly malvidin (53.31%) and its glucosylated form (8.19%)—which are well recognized for their strong antioxidant and anticancer activities [23,24]. Taken together, these findings highlight three distinctive metabolic orientations: a flavonol-rich profile in *T. foenum-graecum*, a precursor-driven phenolic pathway in *L. usitatissimum*, and an anthocyanin dominant profile in *P. miliaceum*. Such metabolic specialization may account for functional complementarities that could be exploited in synergistic formulations for health applications. However, some limitations should be acknowledged. Relative quantification does not provide information on the in vivo bioavailability or actual biological potency of these metabolites. Furthermore, while our findings are consistent with previous studies, additional investigations using cellular and in vivo models are required to validate the functional implications of these compounds.

### 2.3. Mineral Contents

The comparative mineral analysis in Table 3 of *T. foenum-graecum*, *L. usitatissimum*, and *P. miliaceum* revealed marked interspecific variation, indicating distinct nutrient accumulation capacities among these species. *L. usitatissimum* displayed the highest concentrations of Ca (2.443 ± 0.319 mg/g) and Mg (2.868 ± 0.204 mg/g), emphasizing its strong potential as a dietary source of essential structural minerals. This finding aligns with previous reports confirming flaxseed as a rich reservoir of macro- and microelements such as Mg, K, Na, Zn, and Fe, which contribute to bone and metabolic health [45]. *T. foenum-graecum* exhibited higher levels of Na (1.174 ± 0.109 mg/g) and a moderate amount of K (0.536 ± 0.108 mg/g), suggesting a favorable Na/K balance that supports electrolyte and cardiovascular regulation. Although its Ca concentration (2.031 ± 0.103 mg/g) was slightly lower than flaxseed, it remains noteworthy given the species’ known mineral richness. A recent review highlighted fenugreek’s substantial content of potassium, phosphorus, magnesium, and calcium, underscoring its nutritional and therapeutic potential [46]. Compared to both seeds, *P. miliaceum* showed generally lower macromineral levels, particularly for Ca (0.448 ± 0.120 mg/g). However, millets are widely recognized for their high micronutrient density, notably in calcium, iron, zinc, and magnesium, contributing to their value as functional grains in developing regions [47].

Trace element distribution also varied across species. Flaxseed contained the highest Zn concentration (0.430 ± 0.303 mg/g), while fenugreek showed slightly higher iron levels (0.074 ± 0.009 mg/g). These minerals are key cofactors in antioxidant enzymes and play roles in hematopoiesis and immune function. However, the bioavailability of such elements can be limited by the presence of phytate, which chelates divalent cations like Ca^2+^, Zn^2+^, Mg^2+^, and Fe^2+^, reducing absorption efficiency [48].

Overall, the findings delineate three distinct mineral signatures: flaxseed as a Ca, Mg and Zn rich oilseed, fenugreek as a Na, K and Fe dominant legume, and millet as a cereal providing a balanced array of macro and microelements. Their complementary mineral compositions highlight the potential nutritional synergy achievable through combined dietary use. Further investigation should, however, focus on mineral bioavailability and the impact of processing and environmental variables on nutrient stability and absorption efficiency. However, it should be noted that the discussion of mineral bioavailability is based on literature data, as no direct experimental assessment was performed in the present study. However, it should be noted that the discussion of mineral bioavailability is based on literature data, as no direct experimental assessment was performed in the present study. Future studies should also consider the determination of phytic acid content and the calculation of phytate mineral molar ratios to better assess mineral bioavailability. Future studies should also include in vitro anti-inflammatory assays to further explore the broader therapeutic potential of the identified bioactive compounds.

### 2.4. Antibacterial Activity

Table 4 shows that the extract from *T. foenum-graecum* exhibited the most consistent antibacterial activity, producing inhibition zones up to approximately 14.5 mm against *Staphylococcus aureus* at 200 mg/mL, while *L. usitatissimum* and *P. miliaceum* showed more modest effects (maximum ≈ 14 mm and ≈ 10 mm, respectively). The stronger effect of fenugreek aligns with previous reports of its antimicrobial properties [49]. Flaxseed’s weaker yet measurable activity is consistent with its documented antimicrobial potential [50]. These results suggest that fenugreek may be the most promising of the three for antibacterial formulations; however, further characterization—including determination of minimum inhibitory concentrations, compound isolation, and synergy testing with antibiotics is still required.

The minimum inhibitory concentration (MIC) data presented in Table 5 demonstrate that *T. foenum-graecum* exhibited the highest antibacterial potency among the tested plant extracts, with the lowest MIC values recorded against *S. aureus* (62.5 µg/mL) and moderate inhibition of *E. coli* and *Salmonella* (125 µg/mL each). This stronger activity against Gram-positive bacteria agrees with previous findings showing that fenugreek extracts contain saponins and flavonoids capable of disrupting bacterial membranes and inhibiting protein synthesis [51,52]. In addition, the antibacterial activity may involve multiple mechanisms, including disruption of bacterial cell membranes, enzyme inhibition, and induction of oxidative stress, which collectively impair bacterial growth. In contrast, *L. usitatissimum* and *P. miliaceum* showed higher MIC values (125–250 µg/mL), indicating lower antibacterial efficacy. Similar trends have been reported for flaxseed extracts, where phenolic acids and lignans exhibit moderate inhibitory effects depending on solvent polarity and bacterial strain [50]. Although these values are considerably higher than those of standard antibiotics (e.g., ciprofloxacin = 0.25 µg/mL), The antibacterial activity may also be influenced by the presence of mineral elements such as zinc, iron, and copper, which have been reported to contribute to antimicrobial mechanisms.

Overall, the data indicate that *T. foenum-graecum* is the most promising antibacterial candidate, particularly against *S. aureus*, while flaxseed and millet may act as supportive agents with moderate, broad-spectrum effects. Further investigation through purification, synergy, and mechanistic assays is warranted to identify the active constituents and enhance their efficacy relative to standard antimicrobials.

### 2.5. Correlation Matrix

**Figure 1:**
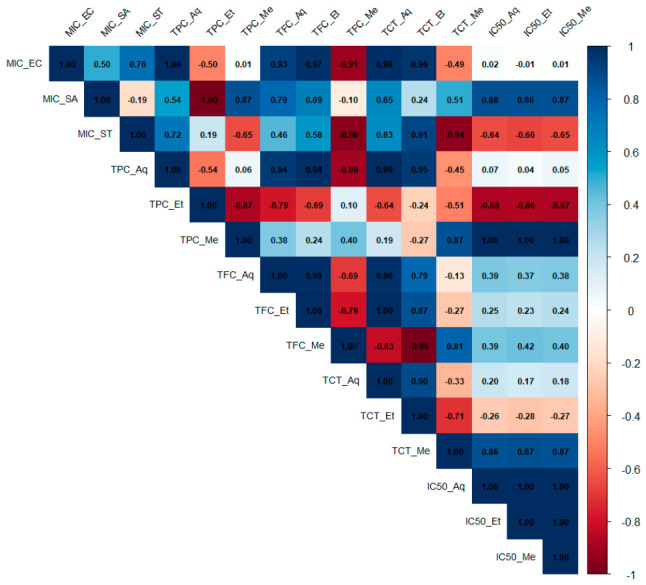
Pearson correlation matrix showing relationships between biochemical activities (TPC, TFC, TCT, IC_50_, and MIC) in aqueous, ethanolic, and methanolic extracts. MIC_EC–*Escherichia coli*; MIC_SA–*Staphylococcus aureus*; MIC_ST–*Salmonella typhimurium*; Aq–Aqueous extract; Et–Ethanolic extract; Me–Methanolic extract.

The correlation matrix (Figure 1) reveals intricate relationships between the phytochemical composition and antibacterial activity (MIC values) of the studied plant extracts. To reflect biological efficacy, MIC values were inverted so that lower MICs represent stronger inhibition. Correlation coefficients were considered statistically significant at *p* < 0.05. Distinct association patterns were observed between MICs and total phenolic (TPC), flavonoid (TFC), and tannin (TCT) contents, as well as antioxidant potential (IC_50_).

Overall, negative correlations between MICs and both phenolic and flavonoid levels indicate that extracts richer in these compounds tend to exhibit stronger antibacterial effects. *T. foenum-graecum*, characterized by particularly high TPC and TFC values, displayed lower MICs especially against *S. aureus* supporting the hypothesis that phenolic compounds play a major role in bacterial growth inhibition. This is consistent with the strong negative Pearson coefficients (r ≈ −0.65 to −0.90) observed between MIC and both TPC and TFC.

Conversely, strong positive correlations between MICs and IC_50_ parameters (r > 0.7 in some cases) indicate that extracts exhibiting weaker antioxidant activity (higher IC_50_ values) generally showed reduced antibacterial potency. This relationship suggests a potential link between free-radical scavenging and antimicrobial mechanisms, possibly mediated by redox-active secondary metabolites.

Strong intercorrelations among phytochemical parameters (TPC, TFC, and TCT; r > 0.8) also indicate a co-accumulation of polyphenolic constituents within the extracts. Such chemical synergy likely contributes to the observed multi-target antibacterial and antioxidant activities.

**Figure 2:**
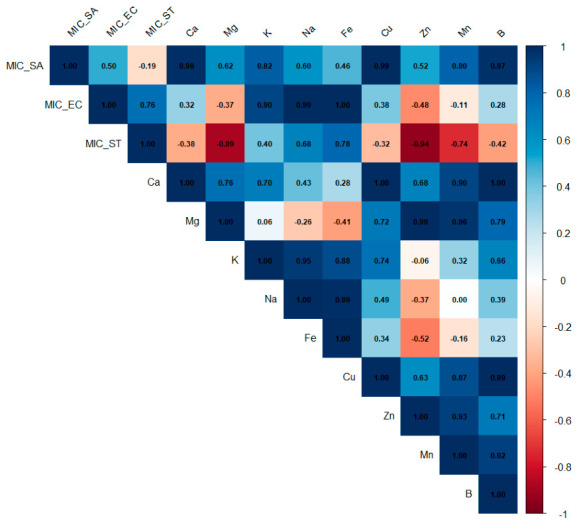
Correlation matrix between antibacterial activity (MIC) and mineral element composition of plant extracts.

Similarly, the correlation matrix (Figure 2) highlights significant relationships between the mineral composition of the extracts and their antibacterial performance. After inversion of MIC values, several macroelements including Ca, K and Na showed strong positive correlations with microbial inhibition (r > 0.70). These elements may contribute to antibacterial activity, possibly through effects on membrane stability or by facilitating the activity of bioactive compounds; however, this interpretation remains tentative and requires further experimental validation.

In contrast, Zn and Mg displayed weak or negative correlations with the adjusted MICs, suggesting limited or even antagonistic effects. The high intercorrelations among Ca, K, and Na indicate a synergistic mineral network that could amplify overall biological responses. Notably, *S. aureus* (MIC_ST) appeared more sensitive to mineral variability than *E. coli* (MIC_EC) or *Salmonella* (MIC_SA), underscoring the strain-dependent nature of the antibacterial effects. It should be noted that correlation does not imply causation, and the observed relationships should be interpreted with caution.

### 2.6. Principal Component Analysis

**Figure 3:**
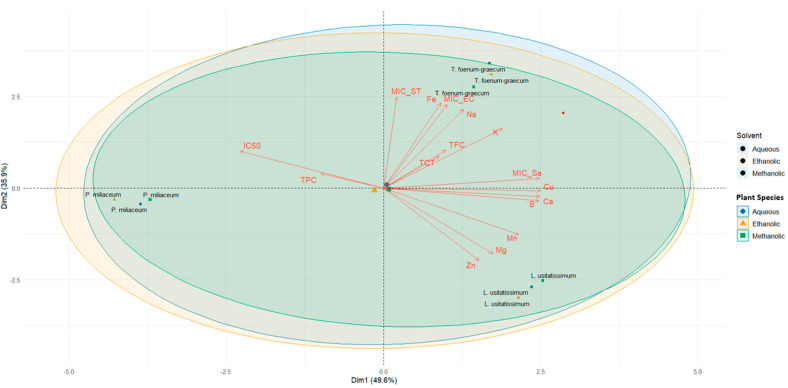
Principal Component Analysis (PCA) showing correlations among biochemical, antibacterial, and mineral parameters in aqueous, ethanolic, and methanolic extracts.

The PCA Figure 3 biplot reveals a pronounced dichotomy in the extract profiles: the first principal component (PC1 ≈ 49.6%) segregates phenolic-rich, antioxidant-active extracts from those with high mineral content and antibacterial potency, while the second component (PC2 ≈ 35.9%) further distinguishes patterns across plant species. On the negative side of PC1, extracts characterized by elevated total phenolic content (TPC) align with low IC_50_ values indicative of strong radical-scavenging capacity, a relationship widely documented in plant matrices [53]. Conversely, on the positive axis, mineral elements such as Ca, Mg, Zn, Fe, and Na exhibit strong loadings together with low MIC values (inverted MICs), suggesting a potential association between mineral composition and antibacterial activity, as also reported in recent multivariate phytochemical-mineral studies [54,55]. However, this relationship should be interpreted with caution, as it remains hypothetical and requires further experimental validation.

The spatial position of *T. foenum-graecum* near these mineral/antibacterial vectors suggests that its extracts exhibit predominantly mineral-mediated antibacterial phenotype; *L. usitatissimum* occupies an intermediate zone associated with elements such as Mn, Zn, and Mg, suggesting moderate antioxidant but strong mineral linked antibacterial behavior; whereas *P. miliaceum* clusters on the phenolic/antioxidant side, indicating high phenolic abundance and radical scavenging capacity but comparatively lower mineral-driven antibacterial activity. A clear solvent-based grouping is also observed: aqueous extracts are mainly associated with the antioxidant/phenolic axis, while ethanolic and methanolic extracts are projected toward the mineral/antibacterial axis. This pattern highlights the influence of solvent polarity on the extraction of different classes of bioactive compounds [53].

Collectively, the PCA highlights that both plant species and extraction solvents play a key role in shaping phytochemical composition and biological activities. Notably, these results suggest a trade-off between antioxidant capacity and antibacterial activity, which should be considered when selecting plant extracts for specific functional applications.

## 3. Materials and Methods

### 3.1. Plant Material

All plant materials used in this study were obtained from a certified herbal practitioner accredited by the Office National de Sécurité Sanitaire des Produits Alimentaires (ONSSA), Rabat, Morocco (33°59′56.071″ N, 6°50′57.055″ W). ensuring the traceability and quality of the samples. The plant materials were already air-dried prior to purchase under traditional conditions. After acquisition, the samples were stored in a dry environment at room temperature until further processing. Before extraction, the samples were finely ground using a professional blender, and the resulting powders were sieved through a 150–180 µm mesh to obtain a homogeneous particle size.

The powdered materials were subsequently subjected to solvent extraction using water, ethanol, and methanol to obtain crude extracts. The resulting extracts were later used for phytochemical, antioxidant, antibacterial, and LC–MS/MS analyses.

The classification and botanical characteristics of the studied plants, including their scientific and common names, families, growth forms, and plant parts used, are summarized in Table 6.

### 3.2. Preparation of Extracts

The dried plant materials were finely ground into homogeneous powders to ensure uniform extraction of their bioactive constituents. For extraction, 10 g of each powdered sample was mixed with 100 mL of solvent either distilled water, ethanol, or methanol. The mixtures were subjected to maceration for 24 h at room temperature with occasional stirring to ensure optimal diffusion of bioactive compounds. The selection of extraction solvents (water, ethanol, and methanol) was based on their different polarities, allowing the extraction of a broad range of bioactive compounds. Water mainly extracts highly polar compounds, ethanol extracts both polar and moderately polar compounds, while methanol is particularly effective for extracting phenolic compounds and other medium-polarity metabolites.

After maceration, the extracts were filtered through Whatman hardened filter paper (AHLESS, Ø125 mm, Clifton, NJ, USA) to remove solid residues. The filtrates were then concentrated under reduced pressure using a rotary evaporator (EVA180, IBX Instruments, Barcelona, Spain), and the obtained crude extracts were stored at +4 °C until further analysis. The resulting extracts were subsequently analyzed for their total polyphenol, flavonoid, tannin, and catechin contents, and their antioxidant activity was evaluated. Additionally, LC–MS/MS-based chromatographic profiling was performed to identify and characterize the major phenolic compounds present in each extract. All extractions were performed in triplicate to ensure reproducibility and reliability of the results.

### 3.3. Determination of Total Polyphenol Content (TPC)

The total polyphenol content of *T. foenum-graecum*, *L. usitatissimum*, and *P. miliaceum* extracts was determined using the Folin–Ciocalteu (FC) colorimetric assay, as previously described [56]. A calibration curve was constructed using a gallic acid standard solution (0.5 g/L), with concentrations ranging from 0 to 200 µg/mL. For the analysis, 200 µL of each extract was combined with 1 mL of 10% (*v*/*v*) Folin–Ciocalteu reagent and allowed to react in the dark for 20 min. Subsequently, 800 µL of sodium carbonate solution (Na_2_CO_3_, 7.5% *w*/*v*) was added, and the mixture was gently mixed. The reaction was then incubated in the dark at room temperature for 3 h.

The absorbance was measured at 765 nm using a UV–Vis spectrophotometer (Peak Instrument C-7200A, Shanghai, China). The total polyphenol content was expressed as micrograms of gallic acid equivalents (µg GAE) per milligram of dry plant material, based on the calibration curve.

### 3.4. Determination of Total Flavonoid Content (TFC)

The total flavonoid content of *T. foenum-graecum*, *L. usitatissimum*, and *P. miliaceum* extracts was evaluated using the aluminum chloride colorimetric assay, according to the method described in [57]. Briefly, 0.25 mL of plant extract was mixed with 1.25 mL of distilled water and 0.075 mL of 5% (*w*/*v*) sodium nitrite (NaNO_2_) solution, and the mixture was allowed to stand for 5 min. After this initial reaction, 0.15 mL of 10% (*w*/*v*) aluminum chloride (AlCl_3_) solution was added. Following an additional incubation period of 6 min, 0.5 mL of 1 M sodium hydroxide (NaOH) was introduced into the mixture. The final solution was incubated at room temperature for 30 min.

The absorbance was then recorded at 510 nm using a UV–Vis spectrophotometer (Peak Instrument C-7200A, Shanghai, China). The total flavonoid content was determined from a quercetin calibration curve and expressed as micrograms of quercetin equivalents (µg QE) per milligram of dry plant material equivalents (µg GAE) per milligram of dry plant material, based on the calibration curve.

### 3.5. Determination of Total Catechin Tannin (TCT)

The content of condensed tannins in the extracts of *T. foenum-graecum*, *L. usitatissimum*, and *P. miliaceum* was determined using the vanillin–HCl colorimetric assay, as described in [56]. Briefly, 50 µL of each extract was mixed with 1.5 mL of 4% vanillin solution prepared in methanol, followed by the addition of 750 µL of concentrated hydrochloric acid (HCl). A reagent blank was prepared under the same conditions. The reaction mixture was allowed to stand at room temperature for 20 min to ensure complete color development. The absorbance was then measured at 500 nm using a UV–Vis spectrophotometer (Peak Instrument C-7200A, Shanghai, China).

The condensed tannin content was quantified using a catechin calibration curve and expressed as micrograms of catechin equivalents (µg CE) per milligram of dry plant material.

### 3.6. Antioxidant Activity

The antioxidant potential of the plant extracts was evaluated based on their ability to scavenge DPPH (2,2-diphenyl-1-picrylhydrazyl) free radicals, following the method described by [58]. In this assay, 0.5 mL of 0.2 mM DPPH solution in ethanol was mixed with 2.5 mL of a diluted extract solution prepared in the same solvent. A blank was prepared under identical conditions, and ascorbic acid was used as the reference standard. The mixtures were vigorously shaken and incubated in the dark for 30 min at room temperature to allow the reaction to proceed.

The decrease in absorbance was recorded at 517 nm using a UV–Visible spectrophotometer (Peak Instrument C-7200A, Shanghai, China). The DPPH radical scavenging activity (%) was calculated using the following formula:

% Inhibition = [(Abs Control − Abs Sample)/Abs Control] × 100

Ascorbic acid was used as a reference standard. It exhibited a strong antioxidant activity with an IC_50_ value of 4.12 µg/mL. All antioxidant measurements were performed in triplicate, and IC_50_ values were calculated from the dose–response curves. Results were expressed as mean ± standard deviation.

### 3.7. Instrument and Chromatography Conditions

The identification and quantification of phenolic compounds were performed using a Thermo Fisher Vanquish UHPLC system (Thermo Fisher Scientific, Waltham, MA, USA) equipped with a binary pump, autosampler, and column oven, coupled to a Thermo Scientific TSQ Altis Triple Quadrupole Mass Spectrometer operating in multiple reaction monitoring (MRM) mode. Chromatographic separation was carried out on a C18 column (150 × 2.1 mm, 3 µm particle size) maintained at 30 °C.

The mobile phase consisted of solvent A (water containing 0.1% formic acid) and solvent B (methanol containing 0.1% formic acid). The elution gradient was optimized to ensure efficient separation of the phenolic compounds: it began with 70% solvent B and 30% solvent A for the first minute, followed by a linear increase to 100% B from 1.01 to 20.00 min to enhance elution of less polar analytes. From 20.01 to 25.00 min, the composition was adjusted to 55% B and 45% A, and then gradually returned to the initial conditions of 70% B and 30% A between 25.01 and 40.00 min for column re-equilibration.

The flow rate was maintained at 0.30 mL/min, with an injection volume of 3 µL [59]. Mass spectrometric detection was conducted in both positive and negative electrospray ionization (ESI±) modes under optimized MRM transitions for each compound The specific LC–MS/MS parameters, including precursor ions, product ions, collision energies, and retention times for the identified phytochemicals, are summarized in Table 7.

Data acquisition and processing were performed using Thermo Xcalibur (version 4.8) and TraceFinder (version 5.1) software. Compound identification was based on the comparison of retention times, mass-to-charge ratios (*m*/*z*), and characteristic fragmentation patterns, using Thermo Scientific software and available literature data. The PubChem Compound Database (NCBI, 2025) was used as a complementary source for retrieving molecular structures and chemical information; however, compound identity was primarily confirmed through LC–MS/MS analytical data.

### 3.8. Minerals Determination

Mineral composition was analyzed following a dry-ashing procedure. In brief, 10 g of dried plant powder were incinerated in a programmable muffle furnace, with the temperature gradually raised from 100 °C to 450 °C over a period of 7 h. After complete ashing, the samples were allowed to cool and treated with 3 mL of distilled water, then evaporated on a hot plate. The residue was re-ashed by heating in the muffle furnace from 200 °C to 450 °C for an additional 2 h, during which 5 mL of concentrated hydrochloric acid (HCl) was added to facilitate mineral release.

Following the second evaporation step, the remaining ash was dissolved in 10 mL of 0.1 mol/L nitric acid (HNO_3_) [60]. The resulting solution was filtered and analyzed for elemental composition using a Varian AA240 graphite furnace atomic absorption spectrometer (GF-AAS, Palo Alto, CA, USA). A total of nine mineral elements were quantified: potassium (K), calcium (Ca), magnesium (Mg), manganese (Mn), copper (Cu), iron (Fe), zinc (Zn), boron (B), and sodium (Na). Method validation was performed using calibration curves established for each mineral element with standard solutions. All calibration curves showed excellent linearity, with correlation coefficients (R^2^) greater than 0.995. The limits of detection (LOD) and limits of quantification (LOQ) were determined for each element, confirming the sensitivity of the analytical method. All measurements were carried out in triplicate to ensure accuracy and reproducibility.

### 3.9. Antibacterial Activity

#### 3.9.1. Agar Diffusion Test

The antibacterial activity of the aqueous extracts of *T. foenum-graecum*, *L. usitatissimum*, and *P. miliaceum* was evaluated using the agar disk diffusion method. The bacterial strains *Escherichia coli* (ATCC 25922), *Salmonella typhimurium* (ATCC 14028), and *Staphylococcus aureus* (ATCC 25923) were cultured on nutrient agar and incubated at 37 °C for 24 h.

Following incubation, bacterial colonies were suspended in sterile saline solution (0.9% NaCl) and adjusted to a 0.5 McFarland standard, corresponding to approximately 1 × 10^8^ CFU/mL.

Sterile blank disks (6 mm in diameter) were impregnated with 10 µL of each aqueous extract and placed onto Müller–Hinton agar plates previously inoculated with the standardized bacterial suspensions. Positive and negative controls were included for validation. The positive controls consisted of Ciprofloxacin (5 µg) for *E. coli*, Gentamicin (10 µg) for *S. typhimurium*, and Oxacillin (1 µg) for *S. aureus* (Oxoid, UK). Disks moistened with distilled water served as negative controls.

The inoculated plates were incubated at 37 °C for 24 h, and antibacterial activity was evaluated by measuring the diameter of the inhibition zones around each disk [61].

#### 3.9.2. Determination of the Minimum Inhibitory Concentration (MIC) of the Extract

The minimum inhibitory concentration (MIC) of the aqueous extracts was determined using a microdilution method in 96-well microplates. The dried extracts were re-dissolved in distilled water prior to analysis. Briefly, 100 µL of Brain Heart Infusion (BHI) medium was added to each well, followed by 100 µL of the extract at an initial concentration of 10,000 µg/mL. Serial twofold dilutions were then performed to obtain final concentrations ranging from 500 to 1.95 µg/mL. A bacterial suspension (10^8^ CFU/mL), prepared from a 24 h culture, was added to each well (10 µL). Positive controls included Ciprofloxacin (Sigma-Aldrich, Merck, CAS: 93107-08-5, Darmstadt, Germany), Gentamicin sulfate (Sigma-Aldrich, Merck, CAS: 1405-41-0), and Oxacillin sodium salt monohydrate (Sigma-Aldrich, Merck, CAS: 7240-38-2, Darmstadt, Germany), while negative controls contained culture medium without extract. The microplates were incubated at 37 °C for 24 h. After incubation, 10 µL of MTT solution (3-(4,5-dimethylthiazol-2-yl)-2,5-diphenyltetrazolium bromide, 0.4 mg/mL in saline) was added to each well, followed by an additional incubation at 37 °C for 10–30 min. Bacterial viability was assessed based on the color change of the MTT reagent, and the MIC was defined as the lowest concentration of extract that inhibited visible bacterial growth [62].

### 3.10. Statistical Data Analysis

All statistical evaluations were carried out using RStudio (version 2025.09.2 Build 418, Posit Software, PBC) coupled with R (version 4.3.1, R Foundation for Statistical Computing, Vienna, Austria). The analysis considered several biochemical and biological parameters, including total phenolic content (TPC), total flavonoid content (TFC), total condensed tannins (TCT), antioxidant activity (IC_50_ DPPH), antibacterial activity (MIC against *E. coli*, *Salmonella typhimurium*, and *Staphylococcus aureus*), and mineral elements (Ca, Mg, Fe, Zn, Mn, Na, K, and B). Variations among plant species and extraction solvents were tested using one-way analysis of variance (ANOVA), with differences considered significant at *p* < 0.05. Prior to ANOVA, assumptions of normality and homoscedasticity were assessed. Pearson’s correlation coefficients were computed to evaluate associations between phytochemical, biological, and mineral variables. To identify patterns and summarize the multidimensional structure of the dataset, Principal Component Analysis (PCA) was performed using the FactoMineR (version 2.9) and factoextra (version 1.0.7) packages. Before performing the PCA, data were standardized using a z-score transformation. To facilitate the interpretation of biological activity parameters, MIC and IC_50_ values were transformed by inversion, so that higher values correspond to stronger antibacterial and antioxidant activities. This approach ensured a consistent direction of variation among all variables included in the multivariate analysis.

Principal components were selected based on the percentage of explained variance, retaining those contributing most significantly to the total variability of the dataset. This multivariate assessment provided an integrated overview of how solvent type and plant species jointly influence chemical composition, antioxidant efficiency, and antibacterial potential.

## 4. Conclusions

This study provides a comprehensive and integrative overview of the biochemical diversity, antioxidant capacity, antibacterial potential, and mineral composition of *T. foenum-graecum*, *L. usitatissimum,* and *P. miliaceum* extracts obtained using different solvents. The novelty of this work lies in the combined application of LC–MS/MS profiling with multivariate statistical analyses (ANOVA, correlation matrix, and PCA), allowing a deeper understanding of the relationships between phytochemical composition, mineral content, and biological activities. Multivariate analyses revealed a clear distinction between phenolic-driven antioxidant activity and mineral-associated antibacterial effects, highlighting the influence of both plant species and solvent polarity. LC–MS/MS profiling further supported these findings by identifying a wide range of bioactive metabolites, including flavonols (kaempferol derivatives), flavones (acacetin, diosmetin), anthocyanins (cyanidin, malvidin, peonidin), and saponins (gypsogenic acid). *T. foenum-graecum* was particularly rich in kaempferol glycosides and isoflavones such as genistin and daidzin, which may explain its strong antioxidant and anti-inflammatory potential. *L. usitatissimum* showed high levels of L-phenylalanine, rutin, and schaftoside, suggesting a profile associated with mineral-related antibacterial activity. *P. miliaceum*, dominated by anthocyanins such as malvidin and cyanidin derivatives, exhibited strong antioxidant potential.

Together, these chemical and statistical analyses demonstrate that each species possesses a distinct phytochemical signature linked to its biological effects. This integrative approach highlights the potential of these plants as sources of multifunctional natural compounds for nutraceutical and pharmaceutical applications. However, these findings should be interpreted with caution, as they are based on in vitro analyses and relative quantification approaches.

Furthermore, this study has certain limitations, including the absence of bioavailability assessment, lack of in vivo validation, and the semi-quantitative nature of LC–MS/MS data. Future research should focus on compound isolation, mechanistic studies, in vivo models, and cellular-based assays to further validate the biological activities of the extracts and better support their potential applications.

## Figures and Tables

**Table 1:** Polyphenolic content (TPC, TFC, TCT) and antioxidant activity of *T. foenum-graecum*, *L. usitatissimum*, and *P. miliaceum* extracts obtained using different extraction solvents.

Species	Solvent	TPC (µg GAE/mg Extract)	TFC (µg QE/mg Extract)	TCT (µg CE/mg Extract)	IC50 (µg/mL)
*T. foenum-graecum*	Aqueous	73.256 ± 0.107	415.240 ± 0.327	34.505 ± 0.012	182.478 ± 0.219
	Ethanol	11.050 ± 0.016	200.440 ± 0.502	45.739 ± 0.011	175.910 ± 0.120
	Methanol	5.600 ± 0.001	28.067 ± 0.007	32.252 ± 0.003	179.307 ± 0.215
*L. usitatissimum*	Aqueous	9.983 ± 0.010	226.630 ± 0.382	7.222 ± 0.003	70.938 ± 0.045
	Ethanol	10.799 ± 0.027	58.053 ± 0.039	0.803 ± 0.001	65.215 ± 0.018
	Methanol	7.702 ± 0.001	65.736 ± 0.013	63.392 ± 0.031	68.826 ± 0.021
*P. miliaceum*	Aqueous	6.447 ± 0.013	113.008 ± 0.291	0.892 ± 0.002	301.371 ± 0.309
	Ethanol	157.438 ± 0.521	11.752 ± 0.012	13.641 ± 0.001	283.700 ± 0.203
	Methanol	3.439 ± 0.003	50.240 ± 0.006	31.937 ± 0.012	292.812 ± 0.402

**Table 2:** Relative quantification of phenolic, flavonoid, and anthocyanin compounds in *T. foenum-graecum*, *L. usitatissimum*, and *P. miliaceum* using LC–MS/MS, expressed as peak area percentage (%).

Molecule	*m*/*z*	Retention Time (min)	Molecular Formula	Compound Class	(%) *T. foenum-graecum*	(%) *L. usitatissimum*	(%) *P. miliaceum*	Bioactivity
**3-glucoside kaempferol**	447.0	3.14	C_21_H_20_O_11_	Flavonol	20.43	-	-	Antioxidant [25]
**3-rutinoside kaempferol**	593.0	18.26	C_27_H_30_O_15_	Flavonol	29.76	-	-	Anti-inflammatory and antioxidant [21]
**Acacetin**	283.2	26.49	C_16_H_12_O_5_	Flavone	-	3.39	2.47	Anti-inflammatory and anticancer [26]
**Caffeoylquinic acid isomer 1**	353.0	18.38	C_16_H_18_O_9_	Phenolic acid	3.25	-	-	Antioxidant and anti-inflammatory [27]
**Caffeoylquinic acid isomer 2**	353.0	18.38	C_16_H_18_O_9_	Phenolic acid	3.25	-	-	Antioxidant and anti-inflammatory [27]
**Cyanidin-3,5-di-O-glucoside**	611.0	37.66	C_27_H_31_O_15_	Anthocyanin	-	-	6.01	Antioxidant [28]
**Cyanidin-3-O-glucoside**	449.1	4.94	C_21_H_21_O_11_	Anthocyanin	18.79	-	3.66	Antioxidant [29]
**Daidzin**	417.1	3.43	C_21_H_20_O_9_	Isoflavone	8.3	-	-	Anti-inflammatory [30]
**Delphinidin**	255.1	28.82	C_15_H_11_O_7_	Anthocyanin	1.61	-	-	Anticancer [31]
**Delphinidin-3-O-glucoside**	465.0	3.24	C_21_H_21_O_12_	Anthocyanin	2.71	1.33	-	Antioxidant [32]
**Dihydroquercetin**	303.0	1.88	C_15_H_12_O_7_	Flavonol	2.12	1.90	-	Antioxidant [33]
**Diosmetin**	299.0	20.63	C_16_H_12_O_6_	Methoxylated flavone	3.10	1.76	18.56	Antioxidant, anti-inflammatory, anticancer, and antimicrobial [34]
**Genistin**	433.1	2.34	C_21_H_20_O_10_	Isoflavone	14.54	-	4.26	Antioxidant and anti-inflammatory [35]
**Gypsogenic acid**	485.33	34.12	C_30_H_48_O_6_	Saponin	-	4.71	18.18	Antioxidant and antimicrobial [36]
**Hesperidin**	611.2	8.28	C_28_H_34_O_15_	Flavanone	-	2.7	-	Neuro-inflammatory activity [37]
**L-Phenylalanine**	166.08	2.44	C_9_H_11_NO_2_	Amino acid	-	78.81	15.64	Antioxidant [38]
**Malvidin**	331.08	27.51	C_17_H_15_O_7_	Anthocyanin	-	-	53.31	Anticancer [23]
**Malvidin-3-O-glucoside**	493.13	9.83	C_23_H_25_O_12_	Anthocyanin	-	-	8.19	Antioxidant [39]
**Nepetin**	317.06	17.51	C_16_H_12_O_7_	Flavone	-	-	6.83	Antidiabetic [40]
**Pelargonidin-3-O-glucoside**	433.11	7.39	C_21_H_21_O_10_	Anthocyanin	-	-	4.26	Anti-inflammatory [41]
**Peonidin-3-O-glucoside**	479.12	8.83	C_22_H_23_O_11_	Anthocyanin	-	-	5.23	Antioxidant [42]
**Rutin**	611.16	8.21	C_27_H_30_O_16_	Flavonol	-	2.68	-	Antibacterial [43]
**Schaftoside**	565.14	2.83	C_26_H_28_O_14_	C-glycosyl flavone	-	3.91	-	Antibacterial [44]

**Table 3:** Elemental Profile of *T. foenum-graecum*, *L. usitatissimum*, and *P. miliaceum* in Dry Matter Basis.

Element	*T. foenum-graecum*	*L. usitatissimum*	*P. miliaceum*
	mg/g Dry Matter		
**Ca**	2.031 ± 0.103	2.443 ± 0.319	0.448 ± 0.120
**Mg**	1.315 ± 0.096	2.868 ± 0.204	1.036 ± 0.902
**K**	0.536 ± 0.108	0.398 ± 0.102	0.294 ± 0.080
**Na**	1.174 ± 0.109	0.765 ± 0.020	0.706 ± 0.017
**Fe**	0.074 ± 0.009	0.054 ± 0.032	0.055 ± 0.031
**Cu**	0.009 ± 0.001	0.010 ± 0.011	0.003 ± 0.008
**Zn**	0.033 ± 0.008	0.430 ± 0.303	0.022 ± 0.071
**Mn**	0.014 ± 0.019	0.020 ± 0.002	0.010 ± 0.020
**B**	0.009 ± 0.002	0.011 ± 0.002	0.003 ± 0.001

**Table 4:** Comparative Antibacterial Activity of Fenugreek and Flaxseed Extracts Against Reference Bacterial Strains.

	Concentration (mg/mL)	Zone of Inhibition (mm)		
		*E. coli* (ATCC 25922)	*Salmonella* (ATCC 14028)	*Staphylococcus aureus* (ATCC 25923)
Antibiotic Control		*26 ± 0.5* ^1^	*23 ± 1.0* ^2^	*22 ± 0.5* ^3^
***T. foenum-graecum***	200	13 ± 1.00	14 ± 0.5	14.5 ± 0.5
	100	8 ± 1.00	9 ± 0.5	13 ± 0.0
	50	Trace (<6)	7.5 ± 0.5	9.5 ± 0.5
	25	NI	NI	7 ± 0.5
	12.5	NI	NI	NI
	6.25	NI	NI	NI
***L. usitatissimum***	200	9 ± 0.0	10 ± 1.0	14 ± 0.00
	100	7.5 ± 1.0	8.5 ± 0.5	11 ± 0.5
	50	NI	Trace (<6)	9 ± 0.0
	25	NI	NI	Trace (<6)
	12.5	NI	NI	NI
	6.25	NI	NI	NI
***P. miliaceum***	200	9 ± 0.50	9 ± 0.5	10 ± 0.0
	100	7.5 ± 0.5	8 ± 0.5	8 ± 0.5
	50	NI	Trace (<6)	Trace (<6)
	25	NI	NI	NI
	12.5	NI	NI	NI
	6.25	NI	NI	NI
**Distilled water**	200	NI	NI	NI
	100	NI	NI	NI
	50	NI	NI	NI
	25	NI	NI	NI
	12.5	NI	NI	NI
	6.25	NI	NI	NI

^1^ Ciprofloxacin (5 µg/disk)–*E. coli*, ^2^ Gentamicin (10 µg/disk)–*Salmonella*, ^3^ Oxacillin (1 µg/disk)–*Staphylococcus aureus*, **NI**: No Inhibition, **Trace (<6)**: Inhibition zone visible but <6 mm.

**Table 5:** Minimum Inhibitory Concentration (MIC) of Aqueous Plant Extracts and Standard Antibiotics Against Reference Bacterial Strains.

	Minimum Hinibatory Concentration (µg/mL)		
	*E. coli* (ATCC 25922)	*Salmonella* (ATCC 14028)	*Staphylococcus aureus* (ATCC 25923)
***T. Foenum-graecum***	125	125	62.5
***L. Usitatissimum***	250	125	250
***P. miliaceum***	250	250	125
**Ciprofloxacin**	0.25	ND	ND
**Gentamicin sulfate**	ND	0.5	ND
**Oxacillin sodium**	ND	ND	1.0
**Negative control (distilled water)**	NI	NI	NI

**ND**: Not determined (not tested on this strain), **NI**: No inhibition observed at all tested concentrations.

**Table 6:** Taxonomic and Morphological Description of the Selected Seeds.

Abbreviation	Scientific Name	Common Name	Botanical Family	Growth Habit	Cultivation Status	Used Part	Appearance
***T*** **. *foenumgraecum***	*Trigonella foenum-graecum*	Fenugreek	Fabaceae	Herbaceous	Cultivated	Seed	Angular, yellow-brown seed
***L*** **. *usitatissimum***	*Linum usitatissimum*	Flaxseed	Linaceae	Herbaceous	Cultivated	Seed	Flat, glossy, dark brown seed
***P.*** ***miliaceum***	*Panicum miliaceum*	Millet	Poaceae	Herbaceous	Cultivated	Seed	Small, round, pale-beige grain

**Table 7:** LC–MS/MS parameters used for the identification and fragmentation of major phytochemical compounds detected in plant extracts.

Compound	*m*/*z* (Precursor)	Major Product Ions	Collision Energy (eV)	Retention Time (min)	Chemical Class
Kaempferol-3-glucoside	447.0	284.9/255.0	25	3.14	Flavonol
Kaempferol-3-rutinoside	593.0	285.0/255.0	30	18.26	Flavonol
Acacetin	283.2	268.9/241.0	35	26.49	Flavone
Caffeoylquinic acid isomer 1	353.0	191.0/179.0	20	18.38	Phenolic acid
Caffeoylquinic acid isomer 2	353.0	191.0/179.0	20	18.38	Phenolic acid
Cyanidin-3,5-di-O-glucoside	611.0	287.0/449.0	25	37.66	Anthocyanin
Cyanidin-3-O-glucoside	449.1	287.0/241.0	25	4.94	Anthocyanin
Daidzin	417.1	255.0/137.0	30	3.43	Isoflavone
Delphinidin	255.1	153.0/137.0	35	28.82	Anthocyanin
Delphinidin-3-O-glucoside	465.0	303.0/157.0	30	3.24	Anthocyanin
Dihydroquercetin	303.0	285.0/179.0	25	1.88	Flavonol
Diosmetin	299.0	284.9/255.0	30	20.63	Methoxylated flavone
Genistin	433.1	271.0/153.0	30	2.34	Isoflavone
Gypsogenic acid	485.3	455.2/425.2	25	34.12	Saponin
Hesperidin	611.2	303.0/151.0	35	8.28	Flavanone
L-Phenylalanine	166.1	120.1/74.1	15	2.44	Amino acid
Malvidin	331.1	316.0/287.0	30	27.51	Anthocyanin
Malvidin-3-O-glucoside	493.1	331.0/287.0	30	9.83	Anthocyanin
Nepetin	317.1	302.9/287.0	25	17.51	Flavone
Pelargonidin-3-O-glucoside	433.1	271.0/151.0	25	7.39	Anthocyanin
Peonidin-3-O-glucoside	479.1	317.0/287.0	25	8.83	Anthocyanin
Rutin	611.2	303.0/271.0	30	8.21	Flavonol
Schaftoside	565.1	283.0/255.0	30	2.83	C-glycosylated flavone

## Data Availability

The original contributions presented in this study are included in the article. Further inquiries can be directed to the corresponding author.
